# miR-1915-3p inhibits Bcl-2 expression in the development of gastric cancer

**DOI:** 10.1042/BSR20182321

**Published:** 2019-05-15

**Authors:** Hong-wei Cui, Wen-yan Han, Li-na Hou, Ling Yang, Xian Li, Xiu-lan Su

**Affiliations:** 1 Department of Cell Biology, Capital Medical University, 10 You An Men Wai Street, Fengtai District, Beijing 100069, China; 2 Clinical Medical Research Center of the Affiliated Hospital, Inner Mongolia Medical University, 1 Tong Dao Street, Huimin District, Hohhot 010050, Inner Mongolia, China

**Keywords:** Apoptosis, Bcl-2, Bcl-XL, Gastric cancer, miR-1915-3p

## Abstract

Many gene expressions changed during the development of gastric cancer, and non-coding RNAs including microRNAs (miRNAs) have been found to regulate cancer progression by participating in the process of tumor cell growth, migration, invasion and apoptosis. Our previous study has identified 29 miRNAs that are highly expressed in gastric cancer stem cells. One of these miRNAs, miR-1915-3p, has shown great potential as a diagnostic and prognostic biomarker for the cancers in liver, colon and thyroid, as well as in immune and kidney diseases. Herein, we found that miR-1915-3p exhibited low expression level in differentiated gastric cancer cell lines and gastric cancer tissues. It was found that the miR-1915-3p inhibited the growth of gastric cancer cells and thus promoted cell apoptosis. We discovered that the expressions of miR-1915-3p were significantly correlated to the lymph node metastasis and overall survival of patients with gastric cancer. Further study showed that there was a negative correlation between miR-1915-3p and Bcl-2 (B cell lymphoma/leukemia-2) expression, suggesting that Bcl-2 was a target gene of miR-1915-3p. Hence, miR-1915-3p possibly contributes to the development and progression of gastric cancer by inhibiting the anti-apoptotic protein Bcl-2. The finding provides a potential therapeutic strategy for gastric cancer.

## Introduction

Gastric cancer is one of the most common malignant tumors of digestive tract and is the leading cause of cancer-related death worldwide [1]. The total number of deaths from gastric cancer accounts for about 10% of all cancer mortality per year [2,3]. It is urgent to improve the early detection and diagnosis, predict the chemotherapeutic efficacy, and to reverse the drug resistance of gastric cancer. Numerous studies reported that the abnormal expression of microRNAs (miRNAs) have been found in some cancer patients and could serve as biomarkers for early detection, treatment and prognosis of gastric cancer [4–10]. Previous miRNA expression profile study has identified 29 miRNAs, such as hsa-mir-338-5p, hsa-mir-3178 and mir-1915-3p that are highly expressed in gastric cancer stem cells [11]. miR-1915-3p is highly expressed in thyroid cancer [12], attenuated oxidative stress responses in hepatocellular carcinoma [13], and associated with immune cell proliferation and senescence [14]. Furthermore, combining the data from TargetScan and miRDB databases, gene ontology, enrichment analysis and pathway analysis revealed that differentially expressed miRNAs are associated with tumor formation and apoptosis-related genes, including Bcl-2 (B cell lymphoma/leukemia-2) family, which is generally recognized as one of the key factors involved in cell apoptosis [15,16].

Bcl-2 gene has more than 20 homologs, which are divided into two major classes based on their mechanisms of action. Some members [e.g. Bcl-2, Bcl-XL (B cell lymphoma/leukemia-XL), MCL-1 (myeloid cell leukemia-1), etc.] are anti-apoptotic, whereas others [e.g. BAX (Bcl2-associated X protein) and Bak] are pro-apoptotic. The synergistic effect of Bcl-2 and BAX can result in the abnormal regulation of proliferation, differentiation and apoptosis in various malignant tumor cells [17,18]. The interaction between miR-1915-3p and Bcl-2 3′-UTR region has been identified and confirmed by bioinformatics analysis and luciferase reporter gene assay. The miR-1915 targeted regulation of Bcl-2 expression in mediating multidrug resistance has been reported in human colorectal cancer cells [19]. However, there is lack of investigation about the relationship between miR-1915-3p and Bcl-2 in the development of gastric cancer. Therefore, the present study aims to explore the expression of miR-1915-3p and its target gene, Bcl-2, in gastric cancer. In addition, the association of miR-1915-3p and Bcl-2 with clinicopathological features and prognosis of gastric cancer patients was evaluated.

## Materials and methods

### Study subjects

A total of 60 patients with gastric cancer tumor resection were recruited from January 2012 to December 2012 at the Gastrointestinal Surgery Department of the Affiliated Hospital of Inner Mongolia Medical University. Patients who received preoperative chemotherapy and targeted therapy were excluded. The average age of patients was 52.67±9.14 years (range 39–78 years). The study protocol was approved by the Ethics Committee of the aforementioned hospital. Written informed consent was obtained from each patient prior to tissue samples collection. All gastric cancer specimens were pathologically confirmed by pathologists. Normal adjacent tissues at least 3 cm away from the tumor tissue were obtained. All patients were followed up for 4–4.5 years.

### Cell lines and reagents

Human gastric carcinoma cell lines BGC-823, SGC-7901, MKN-74 and MKN-45, and GES-1 normal gastric epithelial cell (Cell Bank of Chinese Academy of Science Shang-hai China) were maintained in DMEM high glucose culture medium or modified RPMI-1640 medium (Gibco Industries, Inc. Carlsbad, CA) with 10% fetal bovine serum (Gibco Industries, Inc.), 1% Penicillin/streptomycin solution at 37°C under a humidified atmosphere with 5% CO_2_. The 0.25% Trypsin-EDTA were purchased from Life Technologies (Carlsbad, CA) and from GIBCO (Life Technologies, U.S.A.), respectively. The 25 cm^2^ cell culture flasks, petri dishes (35, 60 and 100 mm in diameter), centrifuge tubes (15 and 50 ml) and storage vials (2 ml) were all obtained from Corning (U.S.A.). Furthermore, micro*ON* miRNA mimic or -control, micro*OFF* miRNA inhibitor or -control were obtained from RiboBio (Guangzhou, China). Amaxa cell line nucleofector kit V was obtained from LONZA (Switzerland). Cell Counting Kit-8 (CCK-8) was obtained from Solarbio life science company (Beijing, China). Annexin V-FITC/PI Apoptosis Detection Kit was purchased from BD company (USA). Trizol reagent, fluorescent dye SYBR Green I, Reverse Transcriptase SuperScript III Reverse Transcriptase, Platinum Taq DNA Polymerase, 100 mm dNTPs and Oligo Synthesis were purchased from Invitrogen (U.S.A.). RIPA protein extraction kit (Pierce, U.S.A.), BCA protein concentration determination kit (Solarbio life science, Beijing, China), mouse monoclonal antibody Bcl-2 and GAPDH were purchased from Santa Cruz Biotechnology, INC. Dylight 800 AffiniPure Goat Anti-Rabbit IgG(H+L) (EarthOx, LLC, San Francisco, CA, U.S.A.).

### Methods

#### Cell culture

Gastric cancer BGC-823, SGC-7901, MKN-74 cells and normal gastric epithelial GES-1 cells were cultured with DMEM medium high sugar (10% fetal bovine serum and 1% double resistance), and incubated at 37°C under a humidified atmosphere of 5% CO_2_. Gastric cancer MKN-45 cell cultured with modified RPMI-1640 medium (10% fetal bovine serum and 1% double resistance), and maintained at 37°C under a humidified atmosphere containing 5% CO_2_. After reaching 80% confluency, the cells were digested with 0.25% trypsin, centrifuged at 900 rpm for 5 min and sub-cultured into new culture flask. The cells in logarithmic growth phase were used for further analysis.

#### miRNA microarray analysis

miRNA expression profiling was performed using Affymetrix GeneChip miRNA 3.0 arrays (Affymetrix, Santa Clara, CA, U.S.A.) as described in the literature [11].

#### Cell transfection

One microliter of 50 nM micro*ON*™ miRNA mimic and the control and 2 µl of 100 nM micro*OFF* miRNA inhibitor and the control were transfected into 96-well cultured gastric cancer cells (1.0 × 10^5^ cells/ml), and then the 96-well culture plate was placed in a 37°C, 5% CO_2_ incubator for 48 h.

#### Cell proliferation determined by CCK-8

One hundred microliter of micro*ON*™ miRNA mimic or control, micro*OFF* miRNA inhibitor or control transfected gastric cancer cells were placed in a 96-well plate, which were pre-incubated in an incubator for 24 h (37°C, 5% CO_2_). The plate was incubated in an incubator for 24 h, 10 μl of CCK8 solution was added to each well, and the plate was incubated in an incubator for 4 h. The absorbance at 450 nm was measured with a microplate reader.

#### Cell apoptosis by Annexin V-FITC/PI APOPTOSIS detection kit

Micro*ON*™ miRNA mimic or control, micro*OFF*™ miRNA inhibitor or control transfected gastric cancer cells were maintained in DMEM high glucose culture medium or modified RPMI-1640 medium with 10% fetal bovine serum, 1% Penicillin/streptomycin solution placed in a 96-well plate at 37°C with 5% CO_2_. After incubating for 48 h, cell apoptosis determined by Annexin V-FITC/PI Apoptosis Detection Kit.

#### Real-time quantitative PCR

Total RNA was extracted from gastric cancer and adjacent normal tissues by using TRIzol^®^ reagent following manufacturer's instructions. The concentration and purity of isolated RNA were determined by NanoDrop 2000. The OD_260_/OD_280_ with ratios in the range of 1.8–2.0 indicate higher levels of RNA purity. Meanwhile, the RNA integrity was evaluated using 1.5% agarose gel electrophoresis. Quantitative PCR (qPCR) reactions were performed in a final volume of 25 μl reaction mixture containing 10 µl of SY-BR super Mix, 2 μl of each primer, 2 µl of template DNA and ddH_2_O. The target gene was amplified by two-step qPCR method. The qPCR cycling conditions were: 95°C for 30 s, followed by 40 cycles of 95°C for 5 s, 60°C for 30 s and 72°C 30 s. All the primer sequences are listed in Table 1.

**Table 1:** The primer sequences used in this work

Gene	Sense	Antisense
miR-1915-3p-RT	GCACTTCAGTGTCGTGGTCAGTGACGGCAATTTGAAGTGCCCCGCCGC	–
miR-1915-3p	CAGACGACCATCAGCCCCAGGGCGACGC	CGCGGATCCATGTCTATTATTACAGATAT
Internal reference 5s	GCCCGATCTCGTCTGATCT	AGCCTACAGCACCCGGTATT
Bcl-2	AGTGGGATGCGGGAGATGT	CGGGCTGGGAGGAGAAGA
Internal reference	CATTCAAGACCGGACAGAGG	ACATACTCAGCACCAGCATCACC
GAPDH		

#### Western blot

The protein of each gastric cancer cells group was extracted using RIPA protein extraction reagent, the protein concentration was determined by BCA kit, subsequently the protein was separated by 12% polyacrylamide gel electrophoresis. iBLOT 2 was transfected, iBind antibody was incubated for 3 h, ODDYSEY far infrared laser scanning was used.

### Bioinformatic analysis and luciferase activity assay

The miR-1915-3p target genes were predicted by miRTarBase (http://mirtarbase.mbc.nctu.edu.tw/php/index.php), miRWalk (http://zmf.umm.uni-heidelberg.de/apps/zmf/mirwalk2/) and DIANA tools (http://diana.imis.athena-innovation.gr/DianaTools/index.php). Luciferase activity was measured using the Dual Luciferase Reporter Assay System (Promega). Firefly luciferase activity was normalized to determine luciferase activity for each group.

### Statistical analysis

Statistical analysis was performed using SPSS 19.0 (SPSS Inc., Chicago, U.S.A.). The expression level of mir-1915-3p was divided into high and low expression groups by using the mean value as a cutoff point. The differences of miR-1915-3p and Bcl-2 expressions between the two sample groups were determined using Wilcoxon test and chi-square test. The correlation between mir-1915-3p and Bcl-2 was evaluated using Pearson's correlation coefficient analysis. *P* values of less than 0.05 were regarded as statistically significant.

## Results

### Expression of miR-1915-3p in gastric cancer cell lines and tissues

**Figure 1:**
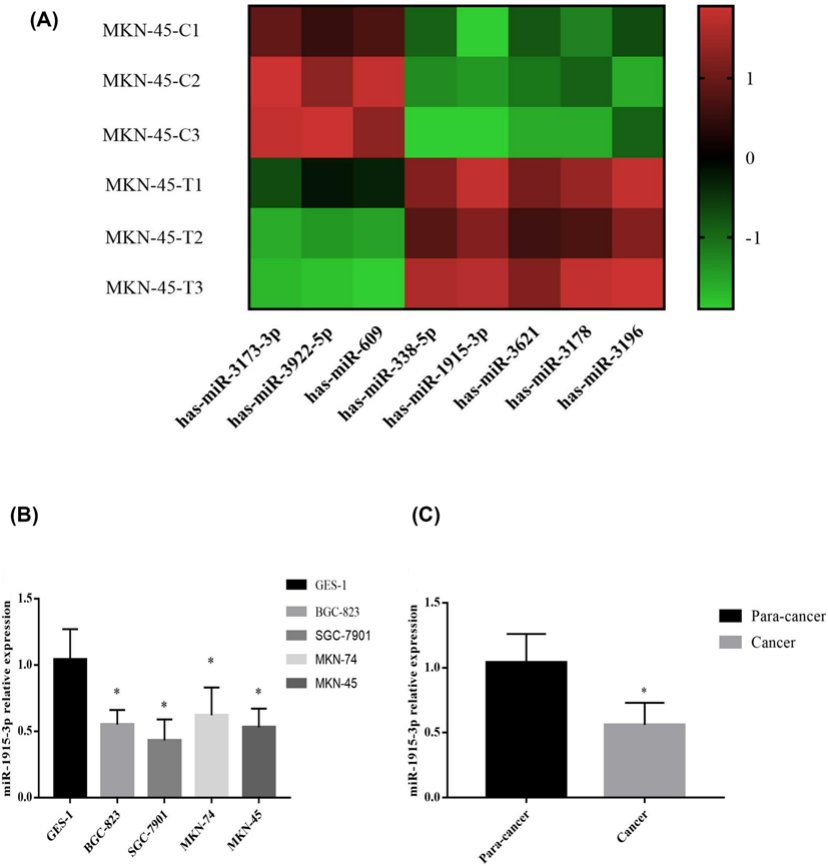
Expression of miR-1915-3p in gastric cancer cell lines (**A**) The expression level of miR-1915-3p in different differentiated gastric cancer compared with GES-1 cells. (**B**) The expression of miR-1915-3p in gastric cancer tissues. (**C**) The expression of several miRNAs before and after treated MKN-45 cells through miRNAs chip.

Previous microRNA microarray results suggest that miR-338-5p, miR-1915-3p, miR-3621, miR-3178 and miR-3196 were down-regulated in MKN-45 cells, whereas the expression of miR-3173-3p, miR-3922-5p and miR-609 were up-regulated (Figure 1A). Cellular level experiments confirmed that the expression level of miR-1915-3p in different differentiated gastric cancer cell lines GBC-823, SGC-7901, MKN-74 and MKN-45 significantly decreased (*P* < 0.05) compared with GES-1 cells (Figure 1B). The expression level of miR-1915-3p in gastric cancer tissues was also lower than that in gastric para-cancer tissues (*P* < 0.05), as shown in Figure 1C. Taken these results together, the expression level of miR-1915-3p was decreased in the gastric cancer cell lines and tissues compared with the normal cells.

### The correlation of miR-1915-3p expression with clinicopathology of gastric cancer

To investigate whether there is a correlation between the expression of miR-1915-3p and clinicopathology of gastric cancer patients, the patients were divided into two groups, i.e. the high miR-1915-3p expression group and the low miR-1915-3p expression group. Patient's clinicopathological features were classified according to tumor size, lymph node metastasis and pathological grading. A significant correlation (*P* < 0.05) between miR-1915-3p expression and lymph node metastasis was observed. However, miR-1915-3p expression showed no statistical correlation with the tumor size and pathological grade (*P* > 0.05), as summarized in Table 2.

**Table 2:** The relationship between the expression of mir-1915-3p and the clinicopathological parameters of gastric cancer

Characteristics	Number	miR-1915-3p		*P* value
		High expression (*n*)	Low expression (*n*)	
Tumor size (cm)				0.631
>3	31	12	19	
<3	29	13	16	
Lymphatic metastasis				0.038
Positive	28	10	18	
Negative	32	20	12	
Pathological stage				0.134
1+2	25	8	17	
3	35	18	17	

### miR-1915-3p expression level with the overall survival of gastric cancer patients

**Figure 2:**
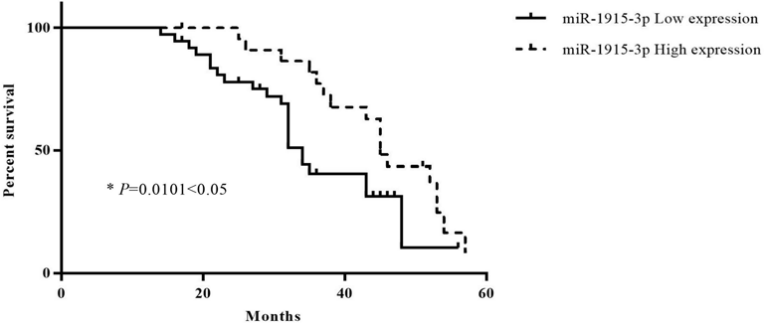
The correlation of miR-1915-3p expression levels with overall survival of gastric cancer patients

The correlation of miR-1915-3p expression level with the survival of gastric cancer patients was estimated by Kaplan–Meier survival method. The Kaplan–Meier survival curve indicates that the gastric cancer patients with high miR-1915-3p expression level (*n* = 37) had shorter overall survival time than those with low miR-1915-3p expression level, as illustrated in Figure 2 and Table 3.

**Table 3:** Gehan–Breslow–Wilcoxon Test for patients with different levels of miR-1915-3p

Items	Median survival (months)	Hazard ratio	95% CI of ratio	Chi square	*P*
miR-1915-3p					
High expression	45	2.051	1.052–3.998	6.621	0.0101
Low expression	34				

### miR-1915-3p inhibited the growth of gastric cancer cells and promoted apoptosis

**Figure 3:**
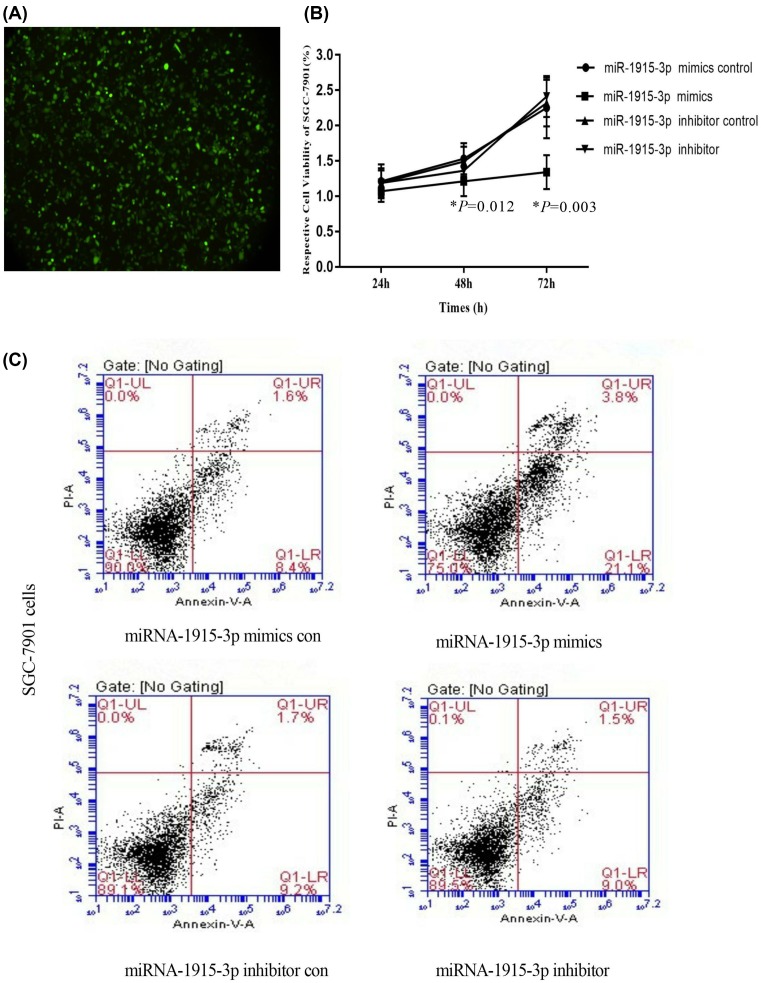
The influence of miR-1915-3p on SGC-7901 cell apoptosis (**A**) GFP was transfected into SGC-7901 gastric cancer cells. (**B**) miR-1915-3p mimics and con, miR-1915-3p inhibitor and -con were transfected into SGC-7901 gastric cancer cells. (**C**) miR-1915-3p mimics and con, miR-1915-3p inhibitor and con were transfected into SGC-7901 gastric cancer cells.

**Figure 4:**
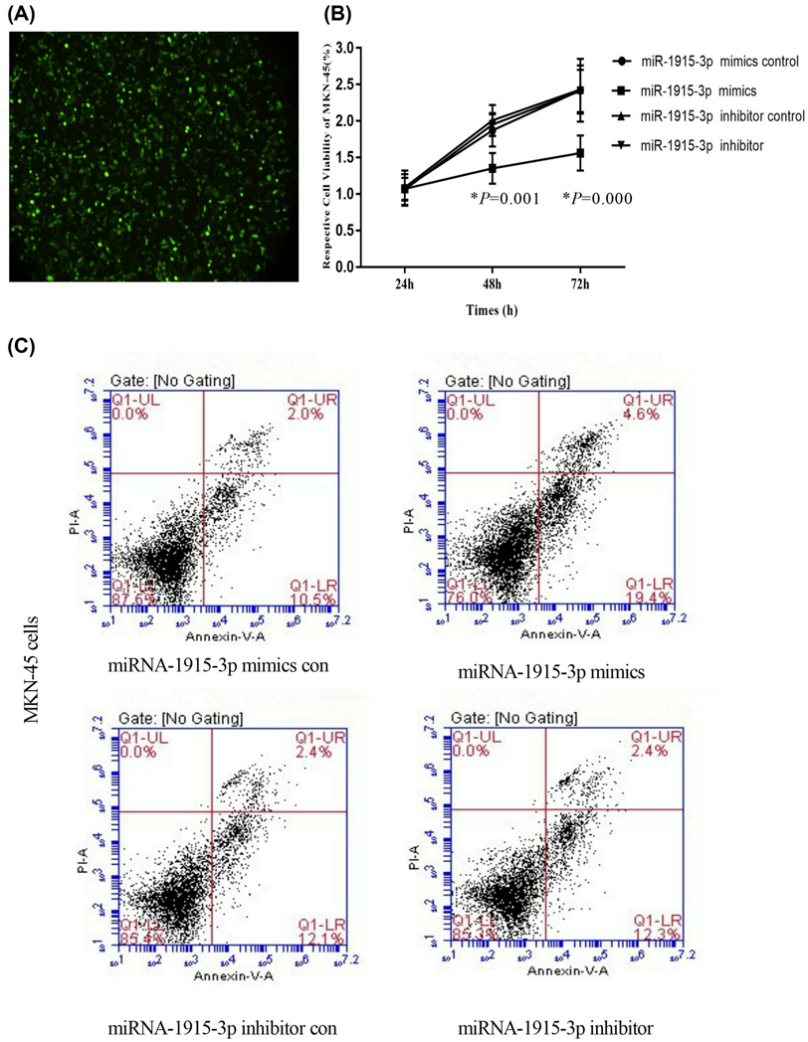
The influence of miR-1915-3p on MKN-45 cell apoptosis (**A**) GFP was transfected into MKN-45 gastric cancer cells. (**B**) miR-1915-3p mimics and con, miR-1915-3p inhibitor and -con were transfected into MKN-45 gastric cancer cells. (**C**) miR-1915-3p mimics and con, miR-1915-3p inhibitor and con were transfected into MKN-45 gastric cancer cells.

The transfection of miR-1915-3p mimics and con, miR-1915-3p inhibitor and con, respectively, into SGC-7901 gastric cancer cells and MKN-45 cells showed that the miR-1915-3p mimics inhibited the growth of both SGC-7901 gastric cancer cells and MKN-45 cells, promoted the apoptosis (*P* < 0.05). However, as shown in Figures 3 and 4, for the miR-1915-3p inhibitor group, the apoptosis was not obvious, and the difference was statistically insignificant (*P* > 0.05) with the gastric cancer cells SGC-7901 and MKN-45 cells.

### Prediction of target gene of the miR-1915-3p

**Figure 5:**
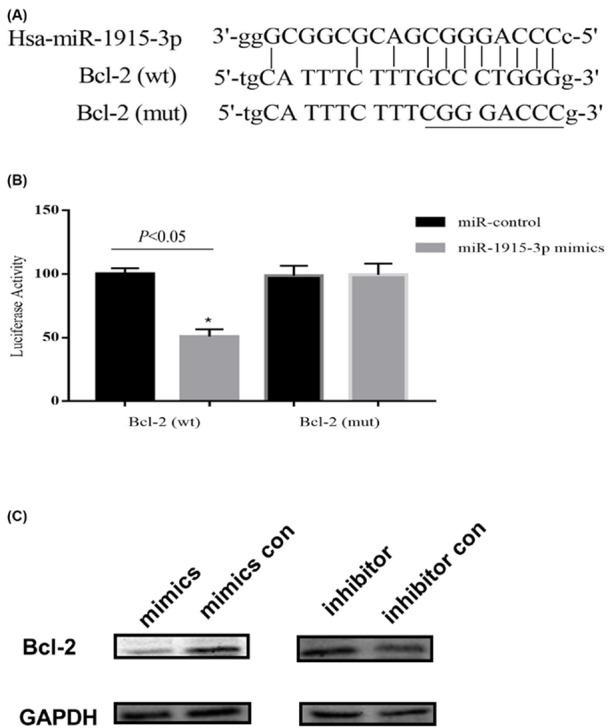
miR-1915-3p target prediction and luciferase activity assay (**A**) miR-1915-3p exhibited identical seed regions, with complementary binding to the 3′-UTR of Bcl-2 (501–507, 1154–1160, 1249–1255, 2109–2115). (**B**) Luciferase activity assay verified the miR-1915-3p complementary binding to the 3′-UTR of Bcl-2. (**C**) Western blot showed the decreased expression of miR-1915-3p in SGC-7901 cells.

miR-1915-3p target genes were predicted using miRTarBase, miRWalk and DIANA tools (Accession ID: MIRT054926; miRNA, hsa-miR-1915-3p; Bcl2, target gene). miR-1915-3p exhibited identical seed regions, with complementary binding to the 3′-UTR of Bcl-2 (501–507 or 1154–1160 or 1249–1255 or 2109–2115) (Figure 5A). To examine whether miR-1915-3p directly targets Bcl-2, a segment of Bcl-2 3′-UTR containing four miR-1915-3p binding sites were cloned into a luciferase reporter system. The plasmid lacking the four binding sites was used as a positive control for luciferase activity assay. The resulting reporter vector was transfected into the SGC-7901 gastric cancer cells. Figure 5B shows that miR-1915-3p mimics inhibited luciferase activity of the cells transfected with Bcl-2 3′-UTR fragment. The luciferase activity remained unchanged when the cells were transfected with Bcl-2 3′-UTR mutant fragment without the miR-1915-3p binding sites (Figure 5B). Western blot result showed that the decreased expression of miR-1915-3p in SGC-7901 cells was concurrent with the overexpression of Bcl-2 protein, suggesting that miR-1915-3p might be subject to post-transcriptional regulation in this context (Figure 5C). Taken together, these results suggested that miR-1915-3p directly inhibits Bcl-2 expression at post-transcriptional level through its 3′-UTR.

### Correlation between miR-1915-3p and Bcl-2 expression

**Figure 6:**
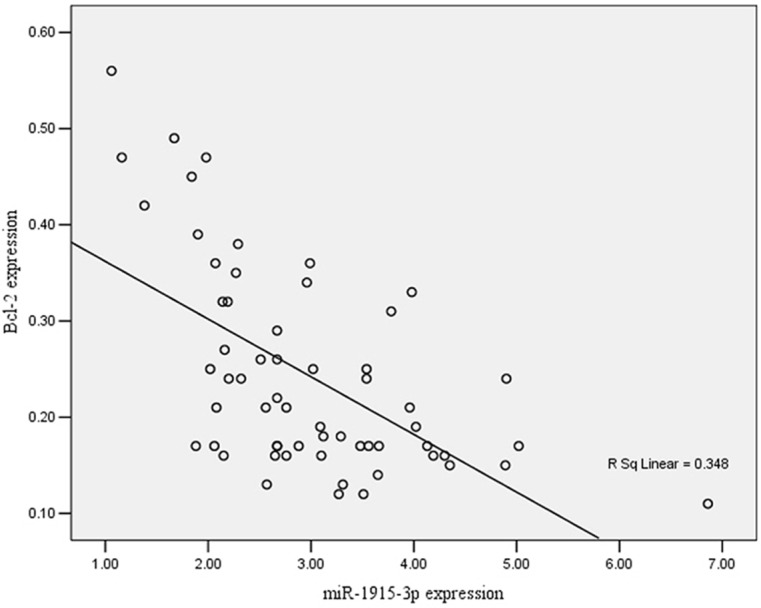
Pearson correlation analysis of the correlation between the expression levels of miR-1915-3p and Bcl-2

Pearson correlation analysis was carried out to explore the correlation between the miR-1915-3p and the expression of Bcl-2. Figure 6 showed that they were negatively correlated to each other (*r* = −0.590) (Figure 6).

## Discussion

Recent advancements of medical technology, surgery and chemotherapy have improved the treatment of gastric cancer patients, but the clinical outcome remains poor, with only 20% of 5-year survival rate [20]. Molecular targeted therapy holds significant promise for the treatment of malignant tumors, including colorectal cancer, lung cancer and breast cancer, by inhibiting tumor proliferation and promoting apoptosis at molecular level [21,22], research that focused on the molecular targeted therapy of gastric cancer is still scarce. Moreover, the etiology and pathogenesis of gastric cancer have not been fully understood. Recently, the biological functions of the non-coding RNA (e.g. miRNA, lncRNA, circRNA, etc.) have been gradually revealed. There is increasing evidence that miRNAs play a pivotal role in regulating cancer cell proliferation, differentiation, apoptosis and other biological processes [23–25]. Dysregulation of miRNAs has been implicated in cancer development and progression, as well as resistance to drug treatment [26,27].

MiR-1915-3p has been explored in bone marrow mesenchymal stem cells, thyroid cancer, kidney disease, Ewing sarcoma, liver cancer, acute myocardial infarction and colorectal cancer. These studies focused on the roles of miR-1915-3p as described below.

1. Diagnostic markers for diseases. Borrelli et al. suggested that high expression levels of miR-1915-3p, as well as other miRNAs with low expression levels might have high diagnostic values and could be applicable for presurgical fine-needle aspiration, especially for indeterminate thyroid nodules [12]. Several novel miRNAs, including miR-1915-3p and several others have been identified to correlate with histopathological lesions and functional markers of kidney damage to facilitate the detection of diabetic nephropathy and lupusnephritis [28].
2. Maintenance of self-renewing stem cells. Sallustio et al. found that miR-1915 and miR-1225-5p seemed to regulate stemness and repair capacity of renal progenitors [29].
3. Reverse multidrug resistance of tumor cells. Xu et al*.* reported that miR-1915 may play a role in the development of multidrug resistance in colorectal carcinoma cells via Bcl-2 targeting [19].
4. Response to DNA damage. Nakazawa et al*.* uncovered a novel mechanism in which p53 could negatively modulate Bcl-2 by targeting miR-1915 [30].
5. Cell proliferation and senescence. Kilpinen et al*.* showed that the expression of miR-1915-3p, miR-1207, miR-3665 and miR-762 in bone marrow mesenchymal stem cells were mainly linked to cell proliferation and senescence [14].

Based on the literatures, the present study tried to explore the correlation and impact of miR-1915-3p on Bcl-2, particularly in gastric cancer. We found that the expression level of miR-1915-3p in gastric cancer cell lines and human gastric cancer tissues, as compared with the control GES-1 cells and para-carcinoma tissues, were lower (Figure 1). The finding was confirmed using miRNAs microarray analysis, which is in accordance with prior report [12] where abnormal miR-1915-3p expression was associated with the infiltrative form of papillary thyroid carcinoma follicular variant.

The low expression level of miR-1915-3p was further examined to understand its effect on gastric cancer. WhenmiR-1915-3p mimics were transfected into SGC-7901 gastric cancer cells and MKN-45 cells, we found that the miR-1915-3p mimics inhibited cancer cell growth and promoted apoptosis. The result suggested that miR-1915-3p may be involved in the progression of gastric cancer, consistent with the observation of a low expression level of miR-1915-3p in gastric cells and tissues. To further understand this result, the miR-1915-3p target gene was predicted using online software and Bcl-2 was found to be a possible target gene, and miR-1915-3p binds to position 1249–1255 of the Bcl-2 3′-UTR. The conclusions were supported by luciferase activity assay result. We further examined the expression of miR-1915-3p and Bcl-2 in gastric cancer tissues and found that miR-1915-3p was negatively correlated with Bcl-2 expression in gastric cancer tissues in which the lower expression level of Bcl-2 corresponds to higher level of miR-1915-3p (Figure 6). Interestingly, significant correlation was also found between miR-1915-3p expression and lymph node metastasis survival of the gastric patient (Table 2), we thus hypothesized that the miR-1915-3p possibly contributes to the development and progression of gastric cancer by inhibiting the anti-apoptotic protein Bcl-2, though the signaling network of miR-1915-3p targeted genes might be extremely complex, especially in the regulation of Bcl-2 apoptotic pathway.

The exploration of the correlation of miR-1915-3p with the other Bcl-2 family proteins, such as BAX, Bim, MCL-1, etc. could be supportive to the current conclusion. Further investigation including the histomorphological change of gastric cancer tissues and larger sample sizes will solidify the understanding of the mechanism underlying miR-1915-3p targeted inhibition to Bcl-2 in the pathogenesis of gastric cancer and exploring the potential therapeutic application of miR-1915-3p.

## Abbreviations

BAX: Bcl-2-associated X protein
Bcl-2: B cell lymphoma/leukemia-2
CCK-8: Cell Counting Kit-8
MCL-1: myeloid cell leukemia-1
miRNA: microRNA
